# CT-to-CT angiography time—an easy target for acute stroke pathway improvement: a retrospective analysis of time from CT to CT angiography in thrombectomy for ischemic stroke

**DOI:** 10.1093/bjr/tqag061

**Published:** 2026-03-16

**Authors:** Tatyana Sarnecki, Marco Mancuso-Marcello, Christos Nikola, Oliver Spooner, Pervinder Bhogal

**Affiliations:** Radiology Department, Addenbrooke’s Hospital, Cambridge University Hospitals NHS Foundation Trust, Cambridge, CB2 0QQ, United Kingdom; Radiology Department, Royal London Hospital, Barts Health NHS Trust, London, E1 1BB, United Kingdom; Stroke Service, Royal London Hospital, Barts Health NHS Trust, London, E1 1BB, United Kingdom; Stroke Service, Royal London Hospital, Barts Health NHS Trust, London, E1 1BB, United Kingdom; Department of Interventional Neuroradiology, Royal London Hospital, Barts NHS Trust and Blizzard Institute, Queen Mary University of London, London, E1 1BB, United Kingdom

**Keywords:** large vessel occlusion, stroke imaging delays, stroke care guidelines, stroke/diagnosis, stroke/therapy, mechanical thrombectomy, NOSIP guideline

## Abstract

**Objectives:**

To assess CT-to-CT angiography (CT-CTA) times at primary stroke centers (PSCs) for patients eligible for mechanical thrombectomy (MT) in acute ischemic stroke and to identify causes of imaging delays.

**Methods:**

This retrospective study analyzed CT-CTA intervals in 200 consecutive patients referred from 18 PSCs in South-East England to a comprehensive stroke center (CSC) (January 2022-March 2023). Times were benchmarked (≤5 min = excellent, ≤10 min = adequate). Inclusion/exclusion following MT guidelines. Confounding variables were analyzed using Welch’s *t*-test and one-way ANOVA. A qualitative survey explored delay causes.

**Results:**

The mean CT-CTA time at PSCs was 62 min (SD 21), vs 1 min (SD 1) at the CSC (*P* < .00001, Hedges’ *g* = 3). Only 9% of PSCs achieved excellent, and 36% adequate, CT-CTA times. No significant differences were found based on time of day, thrombolysis, or NIHSS. However, wide variation existed between PSCs. Survey findings cited technical (eg, lack of CT perfusion, out-of-hours reporting), organizational (eg, scanner access, lack of stroke specialists), and educational (eg, unawareness or dismissal of guidelines) barriers.

**Conclusions:**

CT-CTA delays at PSCs impede timely MT referrals. Improvements in training, infrastructure, and policy (eg, revised SSNAP metrics) are needed for optimizing stroke care pathways.

**Advances in knowledge:**

This study is the first to systematically assess CT-CTA time adherence across PSCs, and it reveals substantial delays and modifiable barriers. It provides actionable insights for optimizing stroke imaging protocols, reinforcing the need for integrated workflows to enhance MT accessibility and outcomes.

## Introduction

Ischemic stroke is a significant cause of mortality and long-term disability worldwide. Mechanical thrombectomy (MT) has revolutionized the treatment of acute ischemic stroke caused by large vessel occlusion (LVO).^1^ However, the time-sensitive nature of reperfusion necessitates prompt diagnosis and intervention. Prompt initiation of MT is crucial, as delays in treatment can result in irreversible brain damage and a poorer prognosis for stroke patients.

**Figure 1:**
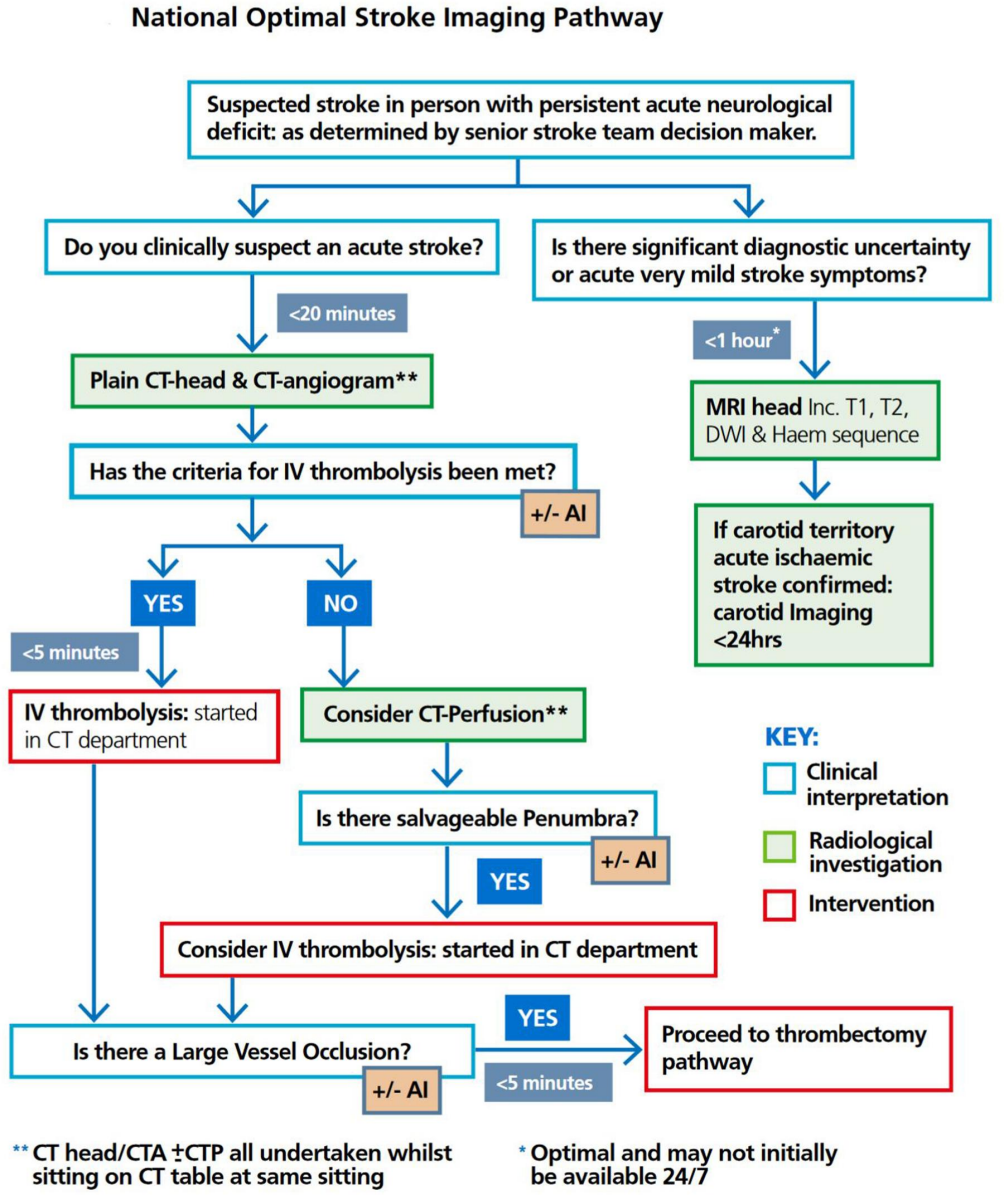
NOSIP from the National Clinical Guideline for Stroke for the United Kingdom and Ireland 2023. Abbreviations: AI = artificial intelligence; CT = computerized tomography; DWI = diffusion-weighted imaging sequence; Haem = hemorrhage identification series; IV = intravenous; MRI = magnetic resonance imaging; T1/T2 = MRI imaging sequences.

To ensure the timely delivery of MT, various guidelines and recommendations have been established by professional medical societies and regulatory bodies.^2–5^ These guidelines encompass different aspects of stroke care, including the assessment of eligible patients, imaging protocols, and recommended maximum time intervals for critical steps such as door-to-needle time or door-in-door-out time. An essential step in evaluating a potential MT candidate is to perform a computed tomographic angiogram (CTA) after excluding a hemorrhage on plain CT, as the CTA not only proves there being a LVO but also provides valuable information about the location and extent of the clot. The CT to CTA time (CT-CTA time) interval is imperative as it directly impacts the subsequent logistics and overall time to treatment; moreover, delays can significantly impact patient outcomes. The importance of this step is reflected by the development of the National Optimal Stroke Imaging Pathway (NOSIP) (Figure 1) in the National Clinical Guideline for Stroke for the United Kingdom and Ireland, which was published May 27, 2021, and updated May 4, 2023.^6^

Evaluating the adherence of hospitals to guidelines regarding CT-CTA time is relevant for several reasons. Firstly, compliance with these guidelines ensures that stroke patients receive appropriate and timely treatment, leading to improved clinical outcomes and lower overall societal costs.^7^ Secondly, monitoring adherence allows healthcare providers and policymakers to identify potential gaps in stroke care delivery and implement targeted interventions to address these issues.^8–12^

In this study, we aim to evaluate the adherence of referring hospitals to the recommended CT-CTA time in the context of MT for acute ischemic stroke. By analyzing real-world data from multiple healthcare institutions, we will assess the average time taken for this critical step and discuss potential factors contributing to any delays observed.

The findings of this study will provide valuable insights into the current practices surrounding CT-CTA time adherence in stroke care. Furthermore, the results can serve as a basis for quality improvement initiatives aimed at optimizing workflow processes and reducing delays in the delivery of MT. Ultimately, our objective is to contribute to the enhancement of stroke care and improve patient outcomes through evidence-based interventions and guidelines.

## Methods

We retrospectively collected the CT and CTA timings of 200 consecutive patients referred from 18 different primary stroke centers (PSCs) from the South-East of England to our London-based comprehensive stroke center (CSC) from January 1, 2022 to March 1, 2023 for MT. Inclusion criteria were patients with acute LVO ischemic stroke that were eligible for MT. For anterior circulation stroke, the inclusion criteria were stroke onset or last seen well under 6 h or less than 24 h with favorable MRI or CT-perfusion mismatch, an ASPECTS score of 6 or above, a proximal clot demonstrated on CTA, and an NIHSS of 6 or more. Exclusion criteria were a premorbid mRS of 3 or worse and presence of severe life-limiting illness. For posterior circulation stroke the inclusion criteria were stroke onset or last seen well less than 12 h and an NIHSS of 10 or more. A premorbid mRS of 2 or worse, and the presence of a life-limiting illness, were reasons for exclusion. The main source of data collection was the standardized MT report form, which was filled out by the receiving INR consultant at the time of the MT. Imaging data from PSCs was not available in all cases to the authors. This is due to the multiple hospitals in our referral network using different PACS systems. Whilst these images are normally uploaded to our native PACS system, this is not always the case. Data on patient characteristics, image and procedure timings, imaging information of the CT and CTA scans obtained at the referring hospital and at the CSC, clot characteristics, details of the treatment, and follow-up information were collected.

We analyzed the time duration between performing CT and CTA imaging at the referring PSCs and compared it to the time of obtaining CTA following CT at our institution, as well as to recommended times in the literature. Whilst as of yet there is no official CT-CTA time target, the National Stroke Service Model considers obtaining CT and CTA in the same sitting best practice,^13^ and the recommended CT initiation to CT interpretation time by the Phase III Target: Stroke is 10-15 min.^14^ We therefore set our cutoff value for an excellent CT-CTA time at ≤5 min and an adequate CT-CTA time at ≤10 min.

We analyzed the data for variations in treatment timings of often cited potential confounding factors, specifically patients presenting in-hours vs patients presenting out-of-hours (OOH), patients who received thrombolysis, and differences based on presenting NIHSS. To establish differences between institutions, we compared the mean and median timings of the PSC that referred ≥8 patients. For this, to minimize small sample bias, we excluded the PSC that referred <8 patients. When analyzing for common confounding factors for all patients, we applied the independent *t*-test assuming unequal variance (Welch’s *t*-test) to determine potential CT-CTA time differences between patients presenting in hours and patients presenting out of hours, and in patients who received thrombolysis and those who did not. All statistical tests were 2-tailed with a significance level set at *P* < .05. A one-way ANOVA was conducted to compare the effect of NIHSS on CT-CTA time. We used the Microsoft Excel Spreadsheet Software from Microsoft 365 (2023) for data recording and statistical analysis. Mean, median, SD, range, 2-sample *t* test with unequal variances, one-way ANOVA, and Hedges’s g effect size were calculated using the integrated Excel data analysis tools.

After the initial presentation of our findings at conferences, to find further factors contributing to the CT-CTA delay at the PSCs, a qualitative questionnaire using Microsoft Forms was sent by email to the PSCs and the CSC in October, November, and December 2024, and a final reminder in January 2025.

## Results

Of the 200 patients referred from PSCs to our CSC hospital, 61% were male, and the average age was 69 years (range 18-95 years). In 21 patients referred from the PSCs, either the CT or CTA time data was not recorded, and these patients were excluded from analysis. From our hospital dataset we excluded 45 data points from analysis because 8 patients went straight to the angiography suite, 33 CTAs were not performed without the reason being recorded, 1 CTA was not performed due to agitation, and 3 data points were excluded due to missing data. This provided us with 179 complete datasets of patients from PSCs and 155 complete datasets for CT-CTA time at our CSC. Of note, since the seconds between CT-CTA were not recorded, the <1 min times of our institution were rounded up to 1 min. The CT-CTA times of <1 min of referring hospitals, however, were rounded down to 0 min. We collected a total of 179 data points on CT-CTA delay time from PSCs with a mean time of 62 min (SD 21 min), a median of 55 min, and a range of 0-232 min. In comparison, in our institution the mean CT-CTA time was 1 min (SD 1 min), the median was 1 min, and the range was 1-55 min in 155 patients who underwent CT and CTA. When analyzing the above times, the independent *t*-test had a *t*-value of 7.58861, and the *P*-value is <.00001. The effect size was very large (Hedges’ *g* = 3). At the referring hospitals, in patients who received multimodal imaging, 40% had a CT-CTA time of ≤5 min and 13% (55% of all patients) ≤10 min. Of the remaining 45%, 80% (34% of all patients) had a delay of ≥30 min, and 30% (13% of all patients) had a delay of ≥60 min.

When subdivided per PSC, only 1 PSC (9%) achieved an excellent mean CT-CTA time. Four PSCs (36%) attained adequate mean CT-CTA timings. When comparing the medians, which will be more statistically robust in these sample sizes, 3f PSCs (27%) had an excellent CT-CTA time and 6 PSCs (55%) an adequate CT-CTA time. An overview of the timings can be found in Table 1. Our institution achieved a CT-CTA time of ≤5 min in 91% of cases with multimodal imaging, and an additional 7% (98% of all patients) had a time ≤10 min.

**Table 1:** Subanalysis of the timings per hospital.^a^

Hospital	No. of referrals	Mean	Median	Fastest	Slowest	First quartile	Third quartile	Interquartile range
1	8	0:06	0:02	0:01	0:35	0:02	0:02	0:00
2	38	0:05	0:03	0:00	1:02	0:02	0:06	0:04
3	15	0:09	0:03	0:00	0:55	0:02	0:11	0:09
4	13	0:10	0:06	0:04	0:42	0:05	0:08	0:03
5	17	0:13	0:07	0:00	1:16	0:05	0:10	0:05
6	25	0:23	0:08	0:00	1:41	0:05	0:36	0:31
7	8	0:26	0:20	0:04	1:10	0:07	0:37	0:30
8	12	0:27	0:25	0:05	1:13	0:09	0:40	0:30
9	17	0:54	0:45	0:08	3:52	0:14	1:09	0:55
10	12	0:50	0:49	0:35	1:37	0:40	0:53	0:13
11	16	0:47	0:57	0:00	1:29	0:40	1:03	0:22

^a^Hospitals with less than 8 referrals were excluded.

There was no significant difference in CT-CTA times between patients presenting in hours (mean 31 min, median 9 min, SD 41 min, range 0-149 min) and patients presenting out of hours (mean 23 min, median 9 min, SD 30 min, range 0-238 min; Welch’s *t* = 1.97, *P* = .99). There was also no significant difference found between patients at the referring hospitals who received thrombolysis (mean 23 min, median 7 min, SD 32 min, range 0-232 min) and the ones who did not (mean 28 min, median 9 min, SD 36 min, range 0-149 min; Welch’s *t* = 1.96, *P* = .41). There was no significant difference of CT-CTA time based on NIHSS (*F* (23, 144) = 1.05, *P* = .41).

Twenty-one percent of the PSCs responded to our qualitative questionnaire, all of whom were consultants. It identified several multifactorial contributors to delays between CT and CTA in the acute stroke pathway. These factors were broadly summarized into technical, organizational, and educational domains.

Technical barriers included an inability to register patients in the hospital’s electronic patient record system while still on the prehospital pathway, despite recent innovations such as pre-alert systems and video triage. Furthermore, the absence of CT perfusion imaging and the unavailability of CTA reporting during OOH periods were highlighted as significant impediments to timely imaging.

Organizational challenges also played a key role. Respondents cited competition for CT access with other clinical specialties, particularly in emergency settings, and the lack of stroke specialist assessments outside standard working hours. Additionally, the absence of financial incentives to refer for MT, as well as criticism of the Sentinel Stroke National Audit Programme (SSNAP) methodology, were noted. Broader systemic issues, including increasing service demand and financial constraints, were seen to exacerbate workflow pressures and contribute to delays.

Lastly, educational gaps were identified. A lack of awareness among non-stroke physicians and allied health professionals regarding the role of CTA in identifying MT candidates was commonly reported. Furthermore, several respondents highlighted that some stroke consultants were either unfamiliar with or dismissive of the NOSIP, undermining the uniform implementation of consensus-driven, guideline-informed imaging protocols.

## Discussion

Mechanical thrombectomy has revolutionized the treatment of patients with ischemic stroke. The initial studies focused on patients treated within 6 h of ictus and with an ASPECT score of ≥6. Further trials have shown positive results for patients treated in the late window, with posterior circulation stroke, and with large core (ASPECT 3-5) with further trials evaluating the potential benefit in more distal occlusions.

Critical to the decision-making process for intervention is the need to conclusively demonstrate the presence of an occlusive thrombus, and in this regard, a CTA is essential. This is unlike the situation regarding IV thrombolysis, which does not require confirmation of an occlusive thrombus on a CT angiogram. As such, it is critical to perform a CT and CTA from the arch to the vertex at the same sitting to prevent unnecessary delays in diagnosis and referral to MT-capable centers. Our results have shown that there are still highly significant delays in obtaining the CTA in a substantial proportion of patients who have access to MT. It is imperative that in-hospital workflows are assessed and optimized to make timely referrals to MT-capable stroke centers, especially where there is no cost associated with optimization of the pathways, as in the case of CT and CTA at the same sitting.

The Sentinel Stroke National Audit Programme (SSNAP) collects a large amount of data for patients with various types of strokes. This was originally set up in the time of thrombolysis to help units monitor and audit their performance with regard to achieving door-to-needle times for IV thrombolysis of <30 min. This is a key performance indicator (KPI) for stroke units delivering thrombolysis in the United Kingdom, and they are graded on this performance. As mentioned above, a requirement for an MT referral is the need for a CTA. Performing a CTA requires approximately 3 more minutes on the CT machine, and this minor delay in delivering thrombolysis will be highly beneficial in identifying patients with LVO and enabling prompt referral. This additional time delay, that would help optimize imaging and detection of LVO in patients, may perversely result in a prolonged time to thrombolysis and a downgrading of your stroke unit. Interestingly, this is something that has occurred in our unit—CT and CTA performed at the same sitting with higher rates of MT but minor worsening of door-to-thrombolysis time because of the additional imaging.

Implementing standardized protocols and quality improvement initiatives has shown promising results in reducing door-to-groin puncture time, with modifications in CT flow producing the largest and most consistent improvements.^11^ The impact of adding routine CTA to stroke protocols is still a subject of debate. Some studies suggest that it does not cause immediate delays,^15^^,^^16^ whilst others demonstrate significant delays, particularly in door-to-needle time when CTA is used as a diagnostic tool.^17^^,^^18^ However, given that there is a substantial incidence of LVO even in patients with mild clinical symptoms,^7^ selective use of CTA in severe cases only may not be ideal. Nonetheless, standardized protocols for patients with suspected LVO, including early CSC notification, CTA on arrival at the PSCs, and cloud-based image sharing, have been shown to reduce time to groin puncture and improve outcomes.^5^ Our qualitative questionnaire revealed that PSCs are aware that their own institutional protocols and their implementations need optimization.

An important factor that needs to be considered when considering the delays in CTA is the radiology workforce. The Royal College of Radiologists (RCR) clinical radiology workforce census in 2022^19^ identified several concerning points, ie, the clinical radiology workforce grew by just 3%. In comparison, demand for diagnostic activity has been rising by over 5% annually, and by around 4% for interventional radiology services. The United Kingdom currently has a 29% shortfall of clinical radiologists, which will rise to 40% in 5 years without action. By 2027, an additional 3365 clinical radiologists will be needed to keep up with demand for services. This fact may well lead to inevitable pushback from overstretched radiology departments when asked to carry out additional CT angiograms. A worry that has already been found to affect PSCs in our region. In order to mitigate these obstacles, we believe that a variety of solutions could be adopted. At a local level there is a need for education between the CSC and PSCs, with knowledge sharing between radiologists at both sites as well as between the stroke physicians and their OOH stand-ins, who will be the first to review the CT and CTA. At a national level the SSNAP metrics should be amended to not inadvertently penalize departments that may delay thrombolysis in order to perform the appropriate imaging to identify LVO. In addition, at the NHS (National Health Service) level, a “best practice tariff” could be instigated for patients who would potentially benefit from MT. This could state that all patients with an NIHSS score of ≥6 and mRS ≤2 should have CT and CTA at the same sitting regardless of the time of onset of stroke. This may have the potential to negate overuse of CTA and the significant increase in radiologist workload at the PSCs.

The importance of reducing delays in stroke care is highlighted by the correlation between CTA delay time and worse clinical outcomes reported by Katz et al.,^20^ emphasizing the need for timely CTA imaging.

Often, part of the total transfer delay is due to factors occurring within individual hospitals,^21^ particularly in the hub and spoke model.^22^^,^^23^ The time from initial CT scan to leaving the primary hospital (door-out) is often overlooked but can significantly impact patient outcomes. Danziger et al. reported a median delay of 93 min in the hub and spoke model.^21^ Similarly, Ng et al. found that the longest component of door-in-door-out (DIDO) time was the CT-to-retrieval-request, with a median of 59.5 min.^24^ This delay is potentially a direct result of CT-CTA delay. Considering that the median CT-CTA time of our PSCs was 55 min, their median door-out time is likely to be even longer than the delays reported in the literature. Our qualitative study revealed technical and organizational examples of causes for these delays, most of which could be properly assessed and addressed with dedicated audits.

The NOSIP guidance has embedded within it the use of AI (artificial intelligence) solutions to assist in the detection of strokes and LVO, and there has been widespread uptake of various solutions within the United Kingdom, with Brainomix currently appearing to be the preferred option within the United Kingdom. Whilst this is a good thing, the benefit of AI augmentation will not be realized if the appropriate imaging is not performed in the first instance. It has already been shown that AI can improve door-in-door-out time by aiding PSCs in their decision-making for onward transport to the thrombectomy center.^25^

Analyzing time delays in stroke treatment at different intervals can provide valuable insights into factors causing delays and help optimize stroke care.^26^^,^^27^ The argument for immediate imaging is that the analysis of the imaging and the organization of further treatment (ie, MT) could be commenced whilst the patient receives thrombolysis, thus optimizing logistics (such as discharging the standby ambulance earlier if a LVO is excluded or notifying the interventional team). The opposing argument is that potential functional brain might be saved by initiating thrombolysis earlier. By recording CT-CTA time, this research question could be answered in the future once sufficient data is collected via registries to analyze the result with a Markov model.

Although there has been a significant increase in the number of MTs being performed, the United Kingdom continues to lag behind our peers in other developed nations with regard to the MT rate with only a 2.4% MT rate seen in the United Kingdom in 2021-2022. There is considerable geographical variation, with London achieving much higher rates of MT than other regions.^28^ Despite this, we have shown that PSCs with 24/7 access to an MT-capable center have not optimized their imaging pathways, and this is causing significant delays to the transfer of patients. Although an oft-quoted reason for lower rates of MT within the United Kingdom is a lack of INRs, this cannot account for the findings reported in this study. Similarly, the centers analyzed in our study have had access to our 24/7 CSC for over 24 months and are yet to optimize their imaging pathways. We believe that an urgent assessment of imaging pathways at PSCs should be performed for the United Kingdom to rapidly address this significant delay causing unnecessary loss of quality-adjusted life years.

## Conclusion

Our study revealed that there is an average delay of 62 min in obtaining a CTA following the initial CT in PSCs that refer to our MT-capable CSC. Furthermore, it demonstrated that a mere 27% of PSCs achieved an excellent median CT-CTA time of ≤5 min, and only 55% an adequate median CT-CTA time of ≤10 min. We discussed potential solutions and areas for further research.
